# A long-term, temporally consistent, gridded daily meteorological dataset for northwestern North America

**DOI:** 10.1038/sdata.2018.299

**Published:** 2019-01-15

**Authors:** A. T. Werner, M. A. Schnorbus, R. R. Shrestha, A. J. Cannon, F. W. Zwiers, G. Dayon, F. Anslow

**Affiliations:** 1 Pacific Climate Impacts Consortium, University of Victoria, Victoria, BC, V8W 2Y2, Canada; 2 Water and Climate Impacts Research Centre, Watershed Hydrology and Ecology Research Division, Environment and Climate Change Canada; 3 Climate Research Division, Environment and Climate Change Canada

**Keywords:** Climate-change impacts, Hydrology

## Abstract

We describe a spatially contiguous, temporally consistent high-resolution gridded daily meteorological dataset for northwestern North America. This >4 million km^2^ region has high topographic relief, seasonal snowpack, permafrost and glaciers, crosses multiple jurisdictional boundaries and contains the entire Yukon, Mackenzie, Saskatchewan, Fraser and Columbia drainages. We interpolate daily station data to 1/16° spatial resolution using a high-resolution monthly 1971–2000 climatology as a predictor in a thin-plate spline interpolating algorithm. Only temporally consistent climate stations with at least 40 years of record are included. Our approach is designed to produce a dataset well suited for driving hydrological models and training statistical downscaling schemes. We compare our results to two commonly used datasets and show improved performance for climate means, extremes and variability. When used to drive a hydrologic model, our dataset also outperforms these datasets for runoff ratios and streamflow trends in several, high elevation, sub-basins of the Fraser River.

## Background & Summary

Climate datasets suitable for trend and extremes analysis enable investigation of the unique challenges facing hydrology today. Temporally consistent datasets better facilitate statistical downscaling and hydrologic modelling by reducing spurious statistical relationships^1,2^. Long-term records are more likely to include observed extreme events that can be used to test modelling frameworks^3–5^ and are better suited to carry out detection and attribution analysis^6^. Currently a long-term, spatially contiguous and temporally consistent gridded meteorological dataset does not exist for northwestern North America (NWNA), 40°N to 72°N and 169°W to 101°W, a region that contains five major drainage basins, the Yukon, Mackenzie, Saskatchewan, Fraser and Columbia. These watersheds have substantial topographic complexity and highly variable climates, which are known to be affected by climate change^7,8^.

To maximize spatial coverage, most gridded meteorological datasets have been based on numerous, often not homogenized, stations of variable record lengths that drop in and out over the timeframe of the dataset^9,10^. These datasets are subject to inhomogeneities that can affect trends or the statistics associated with extremes. Some have attempted to make adjustments so that trends in the gridded dataset match those of long-term stations^11^, while others include a disclaimer stating the data are not intended for use in trend analysis^12^. Homogenized, or temporally consistent, station networks exist in Canada and the US although with a low station density. Hence, they have not been the foundation of the gridded datasets more commonly used with hydrologic modelling or statistical downscaling.

The mountains of western NA provide the majority of the source water to the region’s major rivers^13^. To account for the lack of climate stations at high elevations, interpolation methods often include elevation as a predictor and sometimes include an additional bias correction against a gridded climatology^10,14^. For example, elevation was used as a predictor with the Australian National University Spline (ANUSPLIN) algorithm^15^ to create one of the most commonly used gridded meteorological datasets in Canada^16^, which we refer to as NRCANmet. However, this dataset has known deficiencies for high elevation precipitation^17,18^. One study in New Zealand^19^ showed that using a high-resolution climate normal as a predictor with the spline algorithm, in place of elevation, improved the interpolation of precipitation. Several high-resolution climatologies in NWNA were recently updated to incorporate information from more climate stations^20,21^. One, in British Columbia, was also adjusted against snow course measurements and glacier coverage^20^.

We describe the development of a new, temporally consistent gridded daily meteorological dataset for NWNA covering 1945 through 2012. We refer to this dataset as PCIC meteorology for NWNA, or PNWNAmet. We interpolate a set of long-term homogenized stations with the thin plate spline algorithm using a high resolution, high station density climatology as a predictor. The following will provide a detailed description of the methodology used to create this dataset and its technical evaluation, which includes four main components. First, precipitation, and minimum and maximum temperature from our gridded meteorological dataset and two others commonly used in hydrologic modelling in regions of NWNA, are evaluated with standardize performance measures against an independent climate network for climatology, extremes and variability. Second, their climatologies are compared spatially over NWNA and their climate variability compared temporally by major basin. Third, differences in the water balance between the three datasets is evaluated using observed streamflow through assessment of runoff/precipitation ratios. Fourth, the streamflows simulated by a (calibrated) hydrologic model when driven with the three datasets are evaluated relative to observed streamflow through assessment of streamflow trends.

## Methods

### Primary Data – Stations and Reanalyses

Daily minimum temperature, maximum temperature, and precipitation station records for western Canada were obtained from the second generation of Environment and Climate Change Canada’s (ECCC) Adjusted and Homogenized Canadian Climate Data (AHCCD)^22–24^, a homogenized subset of ECCC’s Historical Climate Data (HCD). Corresponding daily data from the conterminous United States was obtained from the United States Historical Climatology Network-Daily (USHCN-Daily), a stable, long-record subset of the US Cooperative Observer Program (COOP) network only available in the conterminous US^25^. Daily data (not homogenized) in Alaska were obtained from the Global Historical Climatology Network-Daily (GHCN-Daily)^26^.

**Figure 1:**
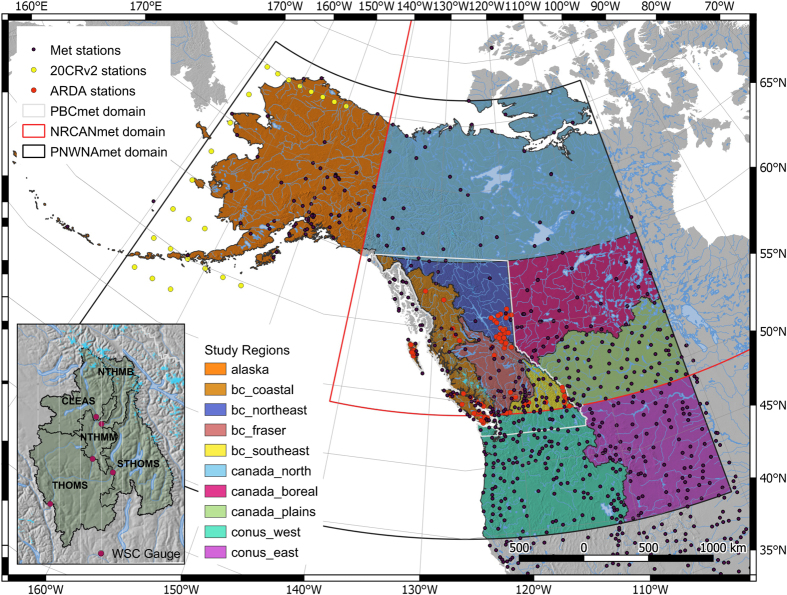
The location of the 20CR, meteorological and ARDA stations, domains of the PNWNAmet, NRCANmet and PBCmet gridded meteorological dataset, sub-regions, and sub-basins of the Thompson (itself a sub-basin of the Fraser River basin; see inset).

To maximize temporal consistency in the dataset, selected stations had to have at least 40 years of complete record (<10% missing days within a year) over the 1945–2012 period. To supplement areas with sparse observations along the periphery of the domain, specifically around the western and northern coasts of Alaska, daily outputs from the 20th Century Reanalysis V2 (20CR2)^27^ were used as virtual stations. This was the only reanalysis spanning the desired period of 1945-2012 for PNWNAmet. Excluding the 20CR2 series, a median of 389 temperature stations (minimum of 322 and maximum of 422) were reported each year; for precipitation, the median number of reporting stations was 442 (minimum of 262 and maximum of 476). Maximum station density in PNWNAmet is given in Fig. 1. Station density is highest in the conterminous United States and southern Canada, decreases with increasing latitude and is lowest in northern Canada, Alaska and northeastern British Columbia.

### Scaling Data – ClimateWNA v5.10

The high-resolution climatology predictor in the thin plate spline interpolation algorithm was derived from ClimateWNA, which is a tool that produces a scale-free, smooth at the boundaries, gridded climatology from a mosaic of disparate climatology products^28–30^. The individual climatologies used, which were the latest available for the provinces, territories and states within NWNA circa 2014, are described in Table 1.

The climatologies were extracted from ClimateWNA to a target resolution of 0.0625° (~6 km) using bilinear interpolation with an elevation adjustment. Elevation was derived from the GEMTED2010 digital elevation model (DEM)^31^, clipped to match the ocean/land boundary. This DEM was selected for its global availability, including areas north of 60°N. Within ClimateWNA all 1961–1990 climatologies were temporally adjusted to the 1971–2000 reference period using anomalies obtained from the CRU ts3.21 dataset^28,32^. Spatial discontinuities between individual climatologies were removed using a gradient merge with a 50-km overlap. This consists of assigning equal weight (50%) to the two overlapping products at the centre of the overlap, then gradually reducing the weights to 0 and 100% at the edges^28^. The result is a single 0.0625°, spatially homogeneous, 1971–2000 monthly precipitation, minimum temperature and maximum temperature climatology that crosses several jurisdictional boundaries.

### Interpolation – Thin Plate Spline

PNWNAmet was created using the trivariate thin plate spline interpolation method, as implemented by Nychka *et al.*^33^, which is similar to the method used in Canada’s NRCANmet dataset^16,34^. As with NRCANmet, minimum temperature, maximum temperature and binary precipitation occurrence and square-root transformed precipitation amounts were interpolated separately on each day, combined, and transformed back to original units. Specifically, values at grid points where the interpolated occurrence exceeded a value of 0.5 were set equal to the interpolated precipitation amount; otherwise, values were set to zero. Instances of maximum temperature less than minimum temperature are possible, and values were simply switched in those cases.

In contrast with NRCANmet, PNWNAmet uses ClimateWNA v5.10 monthly climate normals rather than elevation as the third predictor variable. The rationale is that temporal homogeneity in PNWNAmet is obtained via the use of a relatively small, but fixed number of long record, temporally consistent, daily stations. Additional spatial information is then derived from the underlying climatology. Although the source climatology likely uses the same stations as PNWNAmet, the individual climatologies in Table 1 are constructed from a much larger number of stations, including proxies based on glacier or snow measurements^20,21^. Those climatologies based on the Parameter Regression on Independent Slopes Model (PRISM) also account for additional mechanisms such as temperature inversions and coastal influences. Relying on a mosaic of disparate climatology products is expected to introduce some spatial inconsistency in the final interpolated meteorological fields. However, we feel that any residual spatial inconsistency in the merged ClimateWNA is insignificant compared to any errors that may occur due the interpolation process itself.

In general, thin plate splines are not scale invariant because three covariates, in this case, latitude, longitude and a climate normal appear in a nonlinear term in the interpolation equation. Two estimates of generalization error were used to select optimal scale factors for the climate normal predictor: (1) the generalized cross-validation error, which is calculated as part of the thin plate spline algorithm, and (2) errors based on 10 withheld stations. The 10 stations are drawn from a pool of 50 used by Hutchinson *et al.*^15^ to evaluate NRCANmet across Canada, but restricted to the BC part of the PNWNAmet domain. Minimum and maximum temperature (in °C) and precipitation (in mm) climate normal predictors were scaled by factors of 1/20 and 1/300, respectively. See Boer *et al.*^35^ for full equations and an explanation of how the scaling factors are used. After interpolation, the raw daily minimum temperature, maximum temperature and precipitation surfaces were rescaled so that their climatological monthly means matched the 1971–2000 ClimateWNA v5.10 normals following Hunter and Meetemeyer^36^.

The source data used for PNWNAmet is primarily based on observations collected at fixed times, based on a climate day that does not coincide with the calendar day (i.e. observation times are offset from midnight local time). Offset observations times can result in biases in reported daily maximum and minimum temperature^37^. Changes in the observing day over time also confounds climatic trends^38^ and compiling stations with different observation times can distort areal patterns. Precipitation measurement errors can also affect precipitation trends^39^. We attempt to minimize these effects in the PNWNAmet via the careful selection of source data. For the Canadian AHCCD stations, temperature and precipitation are homogenized to remove any temporal inconsistencies and step changes^22,23^, which are primarily due to station relocations and changes in observing practices^41^. Daily minimum temperature data are further corrected to account for network-wide observation time changes that occurred in 1961 at synoptic stations^23^. In addition, the precipitation data have been corrected for gauge undercatch, evaporation and wetting losses^22^. Although not explicitly homogenized, the USHCN-Daily data are a sub-set of mostly COOP stations that have been selected using several criteria, but primarily based on the degree to which a station maintained a constant observing time^40^. The USHCN-Daily station data was also subject to a number of additional quality assurance checks. Outside the conterminous US, we used GHCN-Daily data, which, although subject to some additional quality assurance^26^, has not undergone any selection or corrections for observation time inconsistencies. Although efforts have been made to select the best available data, the source data are still subject to inconsistencies between observing times within and between networks, which will introduce uncertainties in the resulting interpolated surfaces.

### Data Records

The resulting 1945–2012 Northwestern North America (NWNA) gridded meteorological dataset, PNWNAmet, contains daily precipitation, minimum temperature and maximum temperature gridded to a spatial resolution of 0.0625°. This dataset described is freely available from figshare (Data Citation 1). The data records are stored as four separate variables in a single netCDF file. A duplicate of the dataset is also archived at the Pacific Climate Impacts Consortium’s public and persistent data portal at http://tools.pacificclimate.org/dataportal/gridded_observations/map/.

## Technical Validation

We tested gridded PNWNAmet results and two gridded meteorological datasets commonly used with statistical downscaling and hydrologic modelling in the NWNA region, NRCANmet and PBCmet, against historical station observations from the Agricultural and Rural Development Act (ARDA) network. The ARDA network operated between 1965 and 1991 (https://www.pacificclimate.org/data/bc-station-data) and was only available in BC (Fig. 1). The ARDA station records are sufficiently long to be useful for calculating error statistics, but were not used in any of three gridded meteorological datasets (PNWNAmet, NRCANmet or PBCmet) or the ClimateWNA v5.10 normals. In total, 76 ARDA stations with more than 5 years of data were identified. Stations dropped in and out over the 1965 to 1991 period (not shown). Of these, 20 stations were located at high elevation (>1000 m), which makes the ARDA network particularly well suited for verification in the mountainous regions of BC.

Notable features of the previously mentioned NRCANmet dataset include its use of elevation as the third predictor in the thin plate spline algorithm and source data from ECCC HCD^15^. Furthermore, stations have frequently been added or subtracted over time, peaking in the 1970s with a general decrease towards present^15^. The number of stations active across Canada between 1950 and 2011 ranges from 2000 to 3000 for precipitation and 1500 to 3000 for air temperature^41^. NRCANmet is gridded to a slightly coarser spatial resolution of 0.0833333°.

The PBCmet dataset was generated for BC at a spatial resolution of 0.0625° for 1950–2004 following the methods of Maurer *et al.*^14^. Station data were obtained from multiple national and provincial networks, each with a varying range of quality control and temporal availability. Maximum station density in PBCmet occurred in the 1990s when local networks, such as those available from BC Hydro, the BC Ministry of Forests and BC Ministry of Environment snow pillows, came on line or expanded. The maximum number of stations included 1400 for precipitation and 1130 for temperature^42^. Gridded fields were generated by superimposing interpolated daily station anomalies (relative to their own climatologies) on to a PRISM 1961–1990 monthly climatology. In this fashion, although not acting as a formal covariate (as in PNWNAmet), high-resolution climatology is used to directly influence the spatial structure of the gridded daily fields. Daily anomalies were interpolated using a modified version of the SYMAP algorithm^43^, where a cosine distance function is used in conjunction with a flexible search radius that starts at 50 km and increases until four stations are found (to a maximum radius of 100 km)^44^.The resulting gridded surfaces were then temporally adjusted against homogenized Canadian and US monthly datasets in an attempt to remove artefacts introduced by using a temporally varying station mix^11,45^.

Source data for all the products derives predominantly from national networks with similar quality standards, such as ECCC HCD and AHCCD, COOP, USHCN-daily and GHCN-daily, although PBCmet also uses source data from BC provincial sources including BC Hydro (BCH), and the Automated Snow Pillow (ASP) and Fire Weather (FWN) networks, which may have less stringent quality control. The primary differences in source data between PNWNAmet and the remaining datasets is the absence of short record stations in PNWNAmet, the use of homogenized data in PNWNAmet, and fixed versus varying station mix.

### Evaluation against Station Observations

**Figure 2:**
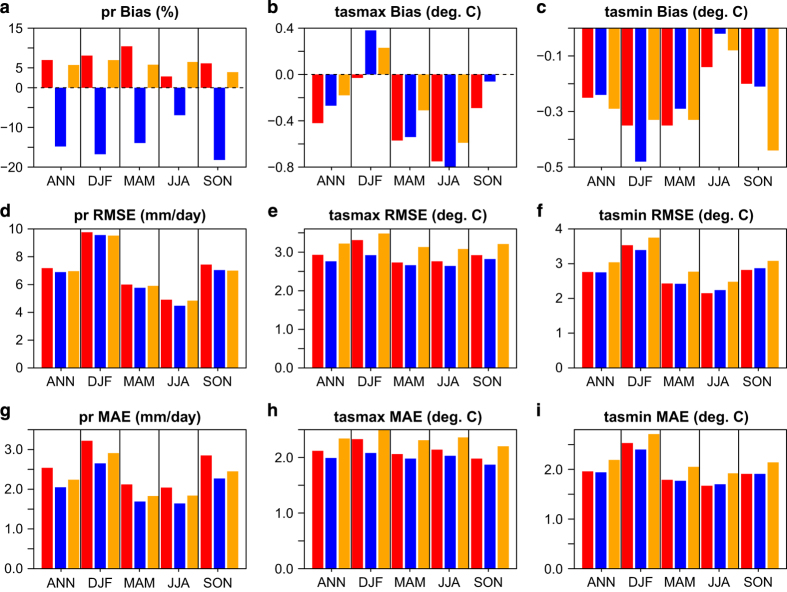
Bias, RMSE and MAE in PNWNAmet (red), NRCANmet (blue) and PBCmet (orange) versus the ARDA network for precipitation (pr), maximum temperature (tasmax) and minimum temperature (tasmin) by season and annually.

Values of mean error (bias), root mean squared error (RMSE), and mean absolute error (MAE) for the three datasets versus the ARDA network are shown in Fig. 2. For precipitation, NRCANmet exhibits a large negative, or dry, bias. Its bias is greatest in fall, followed by winter, then spring, similar to what was found in previous studies^17,18^. PBCmet and PNWNAmet have smaller magnitude precipitation bias than NRCANmet, both being positive. PBCmet shows the lowest magnitude precipitation bias in all seasons except summer. The PNWNAmet dataset has the largest precipitation MAE values of the three datasets, but comparable RMSE values. Given that RMSE puts higher weight on large errors, this suggests poorer performance for low to moderate precipitation intensities for PNWNAmet. Annual bias in maximum temperature is highest for PNWNAmet, whereas it is greatest for PBCmet in minimum temperature. The dataset with the largest temperature bias varies by season for minimum and maximum temperature, although bias in minimum temperature is substantially greater in winter with NRCANmet and in fall with PBCmet. RMSE and MAE are consistently highest for PBCmet annually and for all seasons suggesting a more systematic problem with temperature in this dataset.

It would seem plausible to attribute the large negative bias in NRCANmet, particularly in winter, to the effects of gauge undercatch and loss of trace precipitation, which are known to affect the source data^39,46^. However, if gauge undercatch and trace precipitation strongly affect interpolation accuracy then one would expect PBCmet and NRCANmet to display a similar precipitation bias to ARDA (as both use the same uncorrected source precipitation data). As PBCmet also uses data from additional networks, such as snow pillow sites, found at higher elevations, the effect of precipitation undercatch should dominate this data set even more so than NRCANmet. To the contrary, the similar positive sign and magnitude of the bias between ARDA and both PNWNAmet (using corrected data) and PBCmet (using raw data) suggests that data quality is not the main reason for differences in interpolation bias between NRCANmet and PNWNAmet. We infer instead that interpolation methodology dominates precipitation bias, wherein climatology is a superior predictor for precipitation than elevation (PNWNAmet/PBCmet versus NRCANmet). As all three datasets display similar accuracy for temperature, it is apparent that either elevation or climatology can be used as suitable predictors for temperature.

**Figure 3:**
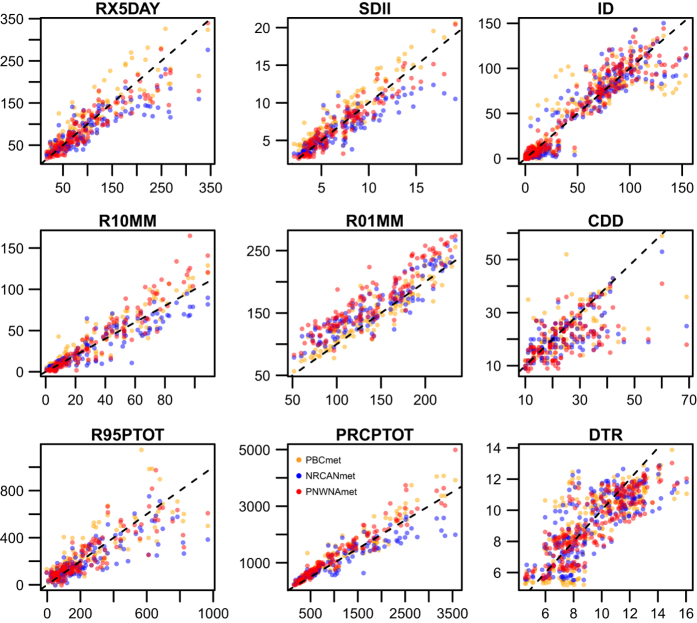
ARDA stations (X-axis) versus modelled (gridded meteorological datasets) values (Y-axis) of nine annual climate indices RX5DAY, SDII, ID, R10MM, R01MM, CDD, R95PTOT, PRCPTOT and DTR.

To evaluate differences in extremes for these datasets, Fig. 3 displays observed (ARDA stations) versus modelled (gridded meteorological datasets) values of nine annual climate indices. Various statistics comparing modelled and ARDA are summarized in Table 2. A dry bias is evident for NRCANmet with the precipitation indices: annual maximum 5-day precipitation (RX5DAY), simple daily wet-day precipitation intensity index (SDII), number of days with precipitation ≥10 mm (R10MM), number of days with precipitation ≥1 mm (R01MM), number of consecutive dry days (CDD), annual total of precipitation from days with precipitation exceeding the 95th percentile (R95PTOT), and annual total precipitation (PRCPTOT). PNWNAmet shows a positive bias in the number of wet days (R01MM), which may stem from the low station density. Large deviations from observed values are noted for PBCmet with the temperature indices: the number of icing days where the maximum temperature <0 deg. C (ID) and mean diurnal temperature range (DTR).

**Figure 4:**
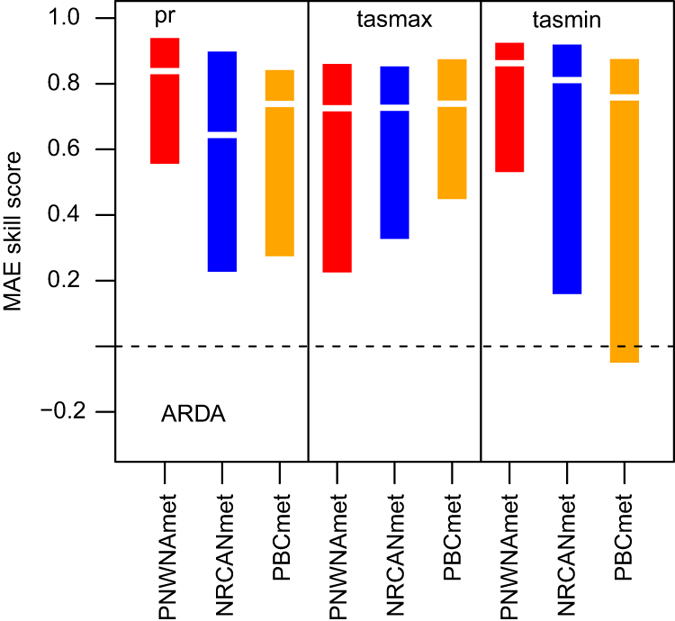
MAE skill scores of cumulative departure series for the gridded meteorological datasets (PNWNAmet, NRCANmet AND PBCmet) versus the observed ARDA stations over British Columbia.

Finally, to assess performance in terms of seasonality and longer-term trends in the datasets, a comparison of cumulative departures of the series from their climatological mean values is made using the distributions of MAE skill scores (MEASS) over the ARDA network for each dataset and variable(Fig. 4), where *MAESS = 1-MAEd/MAEclim*, and MAEd is the MAE of the modelled cumulative departure series relative to the observed cumulative departure series and MAEclim is the same for a simple model consisting of the climatological mean value. MAESS values can range from -Inf to +1, with values greater than 0 indicating skill relative to the naive model. Based on the median and range of MAE skill score the PNWNAmet dataset better resolves the timing of increasing/decreasing periods in the observed series than the NRCANmet and PBCmet datasets, for precipitation and minimum temperature, while PBCmet is slightly better for maximum temperature. PBCmet is particularly weak in minimum temperatures with the lowest median and negative scores. NRCANmet struggles in precipitation. These results reinforce that the methodology for creating the PNWNAmet datasets maintains the timing and magnitude of events found in the observations better than NRCANmet and PBCmet, which rely on a temporally varying station mix.

### Spatial Comparison – PNWNAmet versus NRCANmet

**Figure 5:**
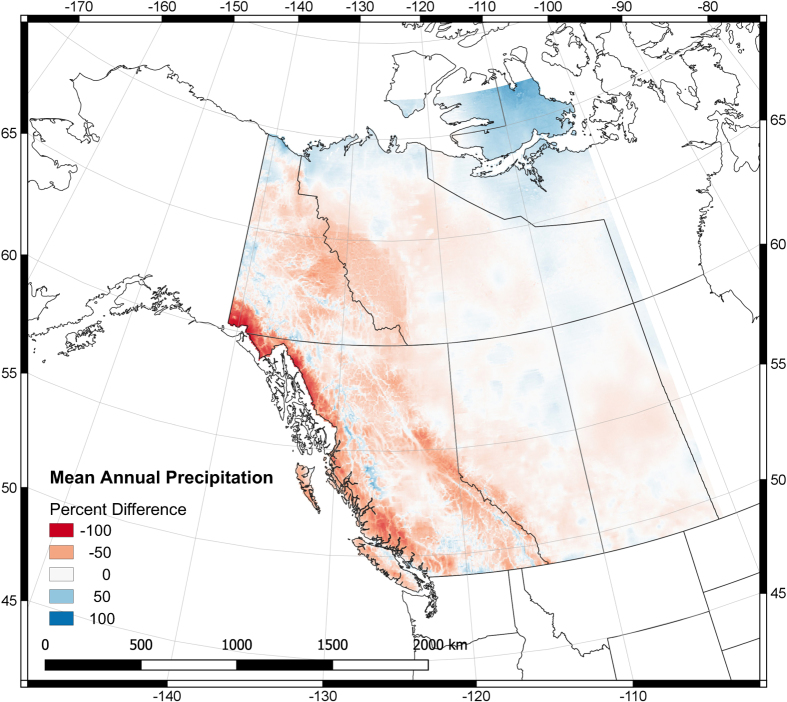
Percent difference in mean annual precipitation for NRCANmet minus PNWNAmet.

Mean annual precipitation, mean annual minimum daily temperature and mean annual maximum daily temperature over 1950 to 2004 are compared between NRCANmet and PNWNAmet over their overlapping region. The dry bias in NRCANmet hinted at by the station comparison in the previous section is shown to be more widespread by the spatial comparison in Fig. 5. The advantage of using climatology as a predictor over elevation for interpolating precipitation is apparent when we see that differences between NRCANmet-PNWNAmet are most pronounced in mountainous regions (Coast and Rocky Mountains and Yukon) and smallest in areas of flat topography (Canadian plains and northern tundra). One notable exception is on Victoria Island, part of the Canadian Arctic Archipelago, where precipitation in NRCANmet is greater than PNWNAmet by up to 76%. Using a hydrologic model Eum *et al.*^46^, found NRCANmet too dry at high elevations. NRCANmet had lower precipitation amounts than other datasets they compared with^17,18^.

NRCANmet is cooler than PNWNAmet in western Canada and warmer in eastern Canada for the long-term average of daily minimum temperature (not shown). NRCANmet is cooler than PNWNAmet in the northwest portion of the region, including northern BC and the Yukon (not shown). Unlike precipitation, temperature differences between NRCANmet and PNWNAmet do not show any spatial coherence with topography.

### Climate Variability

The three gridded meteorological datasets are compared for variability in mean annual precipitation and mean average daily temperature for the ten regions shown in Fig. 1. PNWNAmet is available in all regions, NRCANmet covers all of Canada and PBCmet is only available in British Columbia (BC). Two additional observational datasets are included for precipitation, the Global Precipitation Climatology Centre (GPCC) full data monthly version 2018^47^ and the Variability Analyses of Surface Climate Observations (VASClimO)^48^. Both datasets have global coverage with a spatial resolution of 0.5°. GPCC is a 110-year dataset (1901–2010) recommended for water balance studies, validation of remote sensing based rainfall estimations and verification of numerical models. VASClimO is a 50-Year dataset (1951–2000), which is suitable for climate variability and trend studies. Values are plotted as anomalies from the respective means of the 1950 to 2004 period with loess smoothing (with an 11-year span), except VASClimO, which is available only up to 2000.

**Figure 6:**
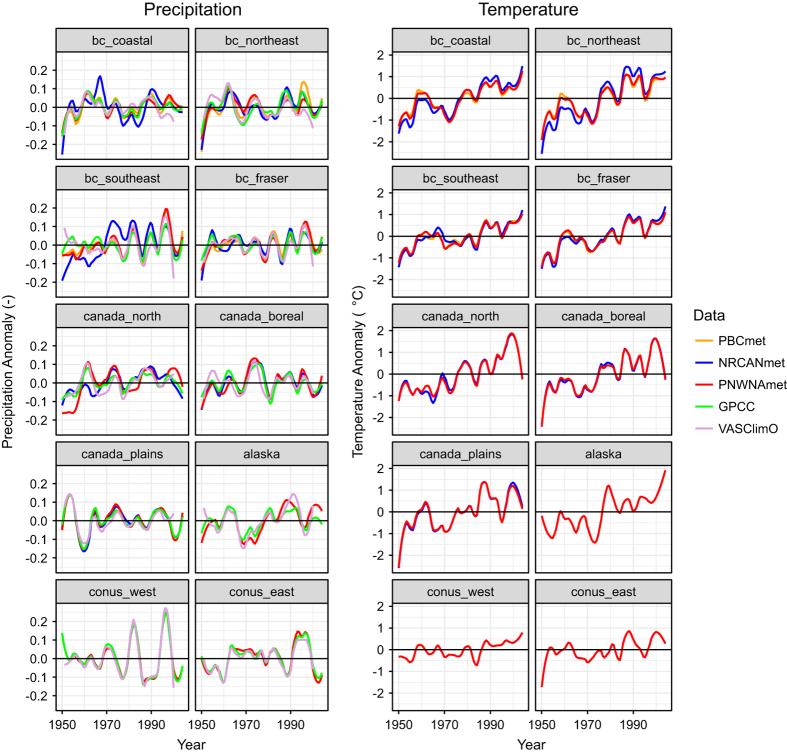
Variability in annual precipitation (pr) and temperature (tas) for 10-year filtered PNWNAmet, NRCANmet, PBCmet, GPCC and VASClimO by region.

Although the broad patterns of inter-annual precipitation variability are similar for all five datasets, notable discrepancies are apparent (Fig. 6). Agreement can be quite poor between all datasets during the early part of the record (prior to 1970), particularly in southeastern BC and northern Canada. In certain regions, specifically coastal and southeastern BC and in northern Canada, NRCANmet exhibits a markedly different temporal evolution than the remaining data sets. The precipitation variability in PNWNAmet tends to match the global observational data sets quite well, except early in the record of the aforementioned regions.

Correspondence of inter-annual variability among the three datasets is much better for temperature than for precipitation. All three datasets also exhibit similar positive trends in annual temperature for all regions, although NRCANmet has a stronger trend than either PBCmet or PNWNAmet in coastal and northeastern BC. Differences between PBCmet and PNWNAmet are, for the most part, indistinguishable.

### Water Balance

To assess basin-wide precipitation accuracy in the gridded climate datasets, we compare precipitation to observed discharge from streamflow gauges. Streamflow integrates precipitation over the drainage area with losses to evapotranspiration, gains due to glacier melt and potential source or sink effects of groundwater. By converting streamflow at the gauge to depth of runoff (R) over a basin, it can be compared to average precipitation (P) over the basin, using the ratio R/P as a surrogate for water balance. R/P values of one or greater would signal that observed runoff is greater than interpolated precipitation, while values of 0.2 or less would suggests strong evaporation rates not likely at these elevations and latitudes^49,50^.

The Thompson above Spence’s Bridge (THOMS) is a major tributary to the Fraser River basin (Fig. 1). It is an ideal setting to investigate the three gridded meteorological datasets for water balance because of its high elevations, unregulated flow, Water Survey of Canada (WSC) observed streamflow records and glacier mass balance data. The THOMS’ drainage area is 55,400 km^2^ and its elevation ranges from 238 m to 3046 m (Table 3). The majority of WSC stations in the THOMS have data available from 1959 to 2013, with the exception of South Thompson River at Chase (STHOM) that starts in 1972. The overlapping period of record for the WSC gauges and three gridded meteorological datasets is 1972 to 2004. Thus, an R/P analysis is conducted over 1971–2000, a standard climate normal period.

Streamflow rates (m^3^s^−1^) were converted to mean annual runoff depth for each sub-basin (mm). A recent study by Beedle *et al.*^51^ looked at 33 glaciers in the Cariboo Mountains, located partially in the headwaters of the THOMS, and found that all glaciers receded during the 1952–2005 period. Rates of retreat and thinning accelerated after 1985. However, glacier cover still remained in this area circa 2005^51,52^. Glacier thinning rates over 1985–1999 were used to adjust average annual runoff^53^. Only in the North Thompson did glacier melt contribute a notable volume to streamflow. Adjusted average annual runoff depth was divided by average annual precipitation depth to give R/P ratios over 1971–2000.

R/P ratios are highest with NRCANmet versus the other gridded meteorological datasets in all sub-basins (Table 4). R/P is greater than one with NRCANmet for the NTHMB despite adjustment of mean annual runoff (R) to account for contributions from glacier melt, while R/P ratios are less than 0.75 for PNWNAmet and PBCmet. R/P ratios in NRCANmet are especially high in the high elevation, headwater catchments of CLEAS, NTHMB and NTHMM. Mean annual precipitation (P) values are lowest for the NRCANmet dataset in all sub-basins. These results confirm the dry bias in NRCANmet at high elevations detected by previous studies^17,18^. Conversely, mean annual R/P values derived from PNWNAmet and PBCmet, with values ranging from 0.53 to 0.73, are considered reasonable for this climate and latitude^49^.

### Hydro-Climatic Trends – Temporal Consistency

Trends in climate data play a major role in determining trends in simulated streamflow. Observed streamflow records can be used as an independent check against modelled streamflow, which in turn assesses trends in the gridded meteorological datasets used to drive the hydrologic model. To check the temporal consistency of the three datasets we continue to use the THOMS as a test basin. The Variable Infiltration Capacity (VIC) hydrologic model^54,55^, commonly applied in this region^5,56,57^, was used to simulate historical streamflow. Simulations were run using VIC version 4.0.7 in water balance mode at a daily timestep (the snow sub-model operated in energy-balance mode at a 3-hour timestep). The computational grid used a spatial resolution of 0.0625° with 500-m elevation bands (with a maximum of five bands per cell) to represent sub-grid topographic variability. The MT-CLIM package in VIC was used to estimate required unobserved variables (such as daily shortwave radiation, longwave radiation, and humidity) and for aggregating daily variables to sub-daily (as required by the snow sub-model)^58^. Wind speed, which is a required input to VIC, was added to the forcing data and was sourced from either NCEP/NCAR Reanalysis 1^59^ (PBCmet and NRCANmet) or NOAA-CIRES 20^th^ Century Reanalysis v2c^27^.

Prior to assessing trend, the VIC hydrologic model was calibrated specifically to each gridded meteorological dataset (NRCANmet, PBCmet and PNWNAmet), while keeping all other parameter fields (vegetation, soil, and elevation) constant. Calibration employed the automated NSGA-II elitist multi-objective genetic algorithm^60^ to optimize a set of five standard runoff generation parameters^61,62^. Over the calibration period (1991–2000) NRCANmet consistently has the largest volume bias (VB) and poorest Nash-Sutcliffe (NSE) and Log NSE coefficients of efficiency performance of the three datasets (not shown). Similar results were obtained for the validation period (1971–1990; Table 5). Large negative streamflow biases with the NRCANmet forcing dataset arise from its negative precipitation bias, which was demonstrated earlier versus the ARDA network (Fig. 2) and via high R/P ratios (Table 4). However, all datasets under predict streamflow volumes, which highlights the difficulty in estimating precipitation in these high elevations areas based on predominately low elevation stations, even when corrected against high-resolution climatologies as with PNWNAmet. Nevertheless, NSE and LNSE scores in all sub-basins, under all three datasets, are sufficiently strong^63^ to proceed with analysing trends in simulated streamflow.

**Figure 7:**
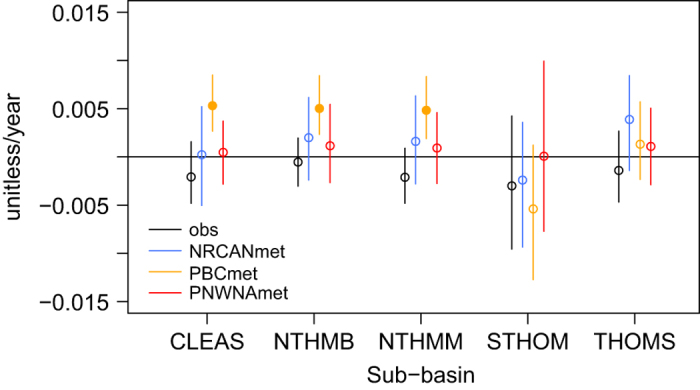
Trends in normalized mean annual observed and simulated streamflow obtained from driving the VIC hydrologic model with NRCANmet, PBCmet and PNWNAmet, over 1959–2004, except STHOM (1972–2004). Circles show trend, filled circles indicate statistically significant trends at a 5% significance level and lines show the range between the upper and lower confidence bounds of the trend.

Trends in mean annual streamflow from the calibrated VIC hydrologic model as driven by the NRCANmet, PBCmet and PNWNAmet gridded meteorological datasets are compared to those for observed streamflow over 1959 to 2004 using the iterative pre-whitening procedure proposed by Zhang *et al.*^64–66^. Trends in annual streamflow are dominated by trends in summer freshet runoff; hence, we omit any seasonal analysis and focus solely on annual streamflow. Streamflow simulated by driving the VIC hydrologic model with the PBCmet has a strong and significant/positive trend, while the observed shows a mild and always non-significant/negative trend for three of five sub-basins, CLEAS, NTHMB and NTHMM (Fig. 7). Additionally, the confidence intervals for the PBCmet trend do not overlap with those for observations in these sub-basins. However, the confidence bounds for trends in streamflow driven by NRCANmet and PNWNAmet do overlap with those for observed in all sub-basins. Thus, some characteristic of the PBCmet dataset is making it unsuitable for use with simulating streamflow trends for the three higher elevation sub-basins.

Trends in annual precipitation, minimum and maximum temperature, and diurnal temperature range were computed on daily data from each gridded meteorological dataset averaged by sub-basin. Similar to streamflow, trends were assessed using the an iterative pre-whitening procedure to correct for autocorrelation^64–66^. These trends are summarized in Table 6. Over the 46 years of analysis, significant increasing trends in mean annual daily precipitation were found in CLEAS, NTHMB and NTHMM only with the PBCmet dataset, while in the STHOM and THOMS only NRCANmet trends were significant. Maximum daily average temperature has increased for all datasets, but trends are significant for the PBCmet dataset only in all sub-basins except STHOM. Maximum temperature trends were highest in magnitude for PBCmet for all sub-basins and lowest in PNWNAmet in all sub-basins, but STHOM. Trends in minimum daily temperature are positive and significant for all three datasets and in all sub-basins. In most cases, minimum temperature trends are also larger in magnitude than are those for the corresponding trend in maximum temperature. As a result, the daily temperature range has been decreasing in most case, although trends are only significant for PNWNAmet.

Trends in annual VIC-simulated values of solar radiation, evapotranspiration and snow accumulation as driven by PBCmet, NRCANmet and PNWNAmet are also summarized in Table 6. Solar radiation exhibits a negative trend for all data sets in all basins. This result is generally consistent with the trends observed for diurnal temperature range, which is used as a covariate for atmospheric transmissivity. However, the variation in trend magnitude between basins and data sets does not correspond strictly to changes in temperature range, as it is affected also by trends in precipitation (which is used as a proxy for cloudiness, such that increasing precipitation would also result in decreasing solar radiation). Evapotranspiration trends are uniformly positive for all data sets and basins and changes are of similar magnitude but mostly insignificant. The magnitude of the evapotranspiration trend is generally the same as the corresponding precipitation trend, except in those instances where the precipitation change is significant and precipitation is increasing at a faster rate than evapotranspiration (e.g. PBCmet for CLEAS, NTHM and NTHM and NRCANmet for STHOM and THOMS). Given the sensitivity of snow accumulation to changes in both temperature and precipitation, simulated March snow water equivalent, which is a surrogate for peak annual accumulation, unsurprisingly shows mixed trends. Large increases occur with PBCmet for CLEAS, NTHMB and NTHMM, but a large decrease occurs for STHOM. PNWNAmet consistently produces decreasing snow water equivalent (or no change for NTHMB), and NRCANmet produces a range of positive and negative changes.

Changes in precipitation (and corresponding trends in snow accumulation) seem to explain the large positive trends in streamflow generated by PBCmet in CLEAS, NTHM, and NTHMB and the negative change produced for STHOM. Large positive precipitation trends, that are much larger than corresponding ET trends, result in increased snow accumulation and discharge (CLEAS, NTHMB and NTHMM). For PBCmet in the STHOM, the trend in ET is positive whereas there is no trend in precipitation, and discharge decreases. For the remaining data sets and basins, any increase in precipitation is partially offset by increasing evapotranspiration and the resulting trends in annual streamflow are statistically insignificant. In these cases, changes in SWE are also generally small and insignificant.

The streamflow trend results for PBCmet indicate that the temporal correction was either ineffective in removing, or possibly exacerbated, the effect of a temporal varying mix of station density and networks. For the PBCmet and NRCANmet datasets, where stations drop in and out over time, the effect of low station density could be more pronounced for the first 20-year period (1958–1977) when the station density was lowest in BC^67^.

## Usage Notes

The PNWNAmet dataset performs comparably well to two gridded meteorological datasets for standardize performance measures against an independent climate network for climatology, extremes and variability. However, it potentially has a greater number of wet days than other gridded meteorological datasets commonly used in the NWNA climate region, which could be due to the lower stations density of this product. It performs better than other datasets in terms of resolving the timing of periods of increasing/decreasing precipitation and minimum temperature in the observed series than the NRCANmet and PBCmet datasets, while PBCmet performs slightly better for maximum temperature. Based on a comparison between PNWNAmet and NRCANmet climatologies, PNWNAmet likely better resolves spatial patterns of precipitation. Furthermore, PNWNAmet better matches decadal scale climate variability of two gridded precipitation products by major drainage basin. Based on a study in the Thompson (a major Fraser River tributary) where all three gridded observational datasets are available, the PNWNAmet and PBCmet datasets are hydrologically consistent based on runoff over precipitation (R/P) ratios where runoff is obtained from observed streamflow. NRCANmet R/P ratios are too high, even exceeding one in one sub-basin. When a hydrologic model is driven with these datasets, the PBCmet driven simulated streamflow results in trends almost opposite from observed in many sub-basins, while NRCANmet and PNWNAmet driven trends are within the confidence bounds of observed.

The temporal and spatial continuity of the PNWNAmet dataset over the Canada/US border at the 49^th^ parallel and from the Yukon into Alaska makes this new dataset ideal for transboundary studies. It also extends far enough east to support hydrologic modelling and statistical downscaling in five major North American drainages, the Yukon, Mackenzie, Saskatchewan, Fraser and Columbia. PNWNAmet appears robust for use with statistical downscaling and hydrological modelling for climatology, trends and extremes over a relatively long-term record (1945–2012). However, where the number of precipitation days is a key determining factor in a physical response of a system, to evaporation or snowmelt, for example, users should be aware of its potential to overestimate the number of days with precipitation. Additionally, in-depth analysis of PNWNAmet has only been conducted for a fraction of its region of availability. Further work is needed to assess PNWNAmet over its entire domain.

## Additional information

**How to cite this article**: Werner, A. T. *et al.* A long-term, temporally consistent, gridded daily meteorological dataset for northwest North America. *Sci. Data*. 6:180299 doi: 10.1038/sdata.2018.299 (2019).

**Publisher’s note**: Springer Nature remains neutral with regard to jurisdictional claims in published maps and institutional affiliations.

## Supplementary Material



## Figures and Tables

**Table 1:** Scaling data (ClimateWNA v5.10) sources.

Source	Years	Resolution	Domain
PRISM	1971–2000	~800 m×800 m	British Columbia, Canada
PRISM	1961–1990	~4 km×4 km	Alberta and Saskatchewan, CA
NRCANmet	1961–1990	~4 km×4 km	Yukon, North West Territories and Nunavut, CA
PRISM	1971–2000	~800 m×800 m	Contiguous US
WorldClim	1961–1990	~1 km×1 km	Alaska, US

**Table 2:** Summary of bias, mean absolute error, correlation and slope of interpolated data versus ARDA.

Index	Dataset	Bias	MAE	Correlation	Slope
RX5DAY	PBCmet	**5.27**	23.63	0.89	**0.88**
	NRCANmet	−15.64	25.04	0.87	0.60
	PNWNAmet	−8.24	**20.15**	**0.92**	0.77
SDII	PBCmet	0.84	1.25	**0.94**	**1.01**
	NRCANmet	−0.62	1.24	0.88	0.63
	PNWNAmet	**−0.12**	**0.92**	**0.94**	0.90
ID	PBCmet	−0.55	11.47	0.90	0.89
	NRCANmet	−1.61	10.30	0.93	0.95
	PNWNAmet	**0.30**	**8.72**	**0.95**	**0.96**
R10MM	PBCmet	4.03	**8.15**	0.94	**1.04**
	NRCANmet	**−3.54**	8.27	0.92	0.81
	PNWNAmet	3.78	8.74	**0.95**	1.24
R01MM	PBCmet	**7.66**	**13.96**	**0.93**	0.90
	NRCANmet	17.36	21.75	0.91	0.82
	PNWNAmet	30.67	32.82	0.90	**0.93**
CDD	PBCmet	**−1.27**	**4.76**	**0.68**	**0.56**
	NRCANmet	−2.93	5.28	0.63	0.45
	PNWNAmet	−4.15	6.19	0.56	0.40
R95PTOT	PBCmet	21.44	86.99	0.82	**0.84**
	NRCANmet	−30.71	78.46	0.82	0.61
	PNWNAmet	**−4.25**	**74.71**	**0.85**	0.76
PRCPTOT	PBCmet	176.00	233.52	**0.96**	**1.07**
	NRCANmet	**−24.10**	240.64	0.92	0.70
	PNWNAmet	193.13	**228.45**	**0.96**	1.08
DTR	PBCmet	**−0.19**	1.27	0.76	**0.69**
	NRCANmet	−0.27	1.25	0.78	0.66
	PNWNAmet	−0.31	**1.06**	**0.85**	0.68
Best performance for a given metric shown with bold, worst shown by underline.					

**Table 3:** Meta data of Thompson sub-basins.

WSC Station ID	Sub-basin Name	WSC Station Name	Drainage Area (km^2^)	Min Elevation (m)	Max Elevation (m)	First Year of Record	Last Year of Record
08LA001	CLEAS	Clearwater River near Clearwater Station	10,300	465	2693	1950	2014
08LB047	NTHMB	North Thompson River at Birch Island	4,490	422	3046	1960	2014
08LB064	NTHMM	North Thompson River at McClure	19,600	358	3046	1958	2013
08LE031	STHOM	South Thompson River at Chase	15,800	344	2749	1972	2013
08LF051	THOMS	Thompson River Near Spence’s Bridge	55,400	238	3046	1951	2013

**Table 4:** Runoff versus precipitation in the Thompson sub-basins.

Sub-basin Name	Total Runoff (mm)	Glacier Runoff (mm)	Mean Annual Precipitation (mm)			Adjusted R/P (unitless)		
			NRCAN met	PNWNA met	PBCmet	NRCAN met	PNWNA met	PBCmet
CLEAS	693	0	775	1018	1016	0.89	0.68	0.68
NTHMB	1050	26	984	1475	1394	1.04	0.69	0.73
NTHMM	693	6	810	1063	1039	0.85	0.65	0.67
STHOM	632	0	904	1029	970	0.70	0.61	0.65
THOMS	451	0	718	853	829	0.63	0.53	0.54

**Table 5:** Nash–Sutcliffe coefficient of efficiency (NSE), NSE of log-transformed discharge (LNSE) and volume bias (VB) for five Water Survey of Canada stations in the Thompson sub-basin of the Fraser River Basin over 1971–1990.

Basin	PBCmet										PNWNAmet			NRCANmet		
	NSE	LNSE	VB	NSE	LNSE	VB	NSE	LNSE	VB							
CLEAS	**0.86**	0.83	**−11.9**	0.79	**0.87**	−17.8	0.70	0.64	−33.2
NTHMB	0.78	0.81	−10.0	**0.80**	**0.88**	**−9.0**	0.67	0.62	−37.3
NTHMM	**0.86**	0.86	**−11.0**	0.82	**0.89**	−14.6	0.74	0.73	−31.5
STHOM	**0.92**	0.80	**−8.9**	0.89	**0.88**	−10.3	0.89	0.77	−14.9
THOMS	**0.93**	0.91	**−4.6**	0.89	**0.92**	−8.3	0.87	0.84	−19.9
Best performance for a given metric shown with bold, worst shown by underline.									

**Table 6:** Trend in annual total precipitation, daily maximum and minimum temperature, daily temperature range, daily solar radiation, total evapotranspiration and March snow water equivalent over Thompson sub-basins from 1959 to 2004 (change per 46 years).

Variable (units)	Data Set	Trend by Basin						
		CLEAS	NTHMB	NTHMM	STHOM	THOMS		
Precipitation (mm)	PBCmet	**196**	**283**	**192**	2	54
	NRCANmet	38	49	69	**162**	**108**
	PNWNAmet	25	34	27	73	46
Maximum Temperature (°C)	PBCmet	**1.0**	**1.1**	**1.1**	0.9	**1.0**
	NRCANmet	0.9	0.7	0.8	0.3	0.7
	PNWNAmet	0.3	0.2	0.2	0.6	0.5
Minimum Temperature (°C)	PBCmet	**1.2**	**1.0**	**1.2**	**1.4**	**1.5**
	NRCANmet	**1.2**	**1.8**	**1.4**	**1.7**	**1.6**
	PNWNAmet	**1.2**	**1.0**	**1.2**	**1.4**	**1.5**
Daily Temperature Range (°C)	PBCmet	−0.2	0.1	−0.1	−0.4	−0.4
	NRCANmet	0.0	−0.7	−0.4	−1.1	−0.7
	PNWNAmet	**−0.9**	**−0.8**	**−0.9**	**−0.7**	**−0.8**
Solar Radiation (W m^−2^)	PBCmet	**−13**	−11	−12	**−13**	**−12**
	NRCANmet	**−10**	**−7**	**−10**	**−12**	**−12**
	PNWNAmet	**−11**	**−12**	**−11**	**−14**	**−11**
Evapotranspiration (mm)	PBCmet	24	21	28	28	34
	NRCANmet	**45**	28	**43**	35	**57**
	PNWNAmet	17	10	15	22	21
March Snow Water Equivalent (mm)	PBCmet	68	**148**	67	−68	−26
	NRCANmet	−22	15	−1	44	−10
	PNWNAmet	−14	0	−11	−20	−22
Bold indicates statistically significant change at the 5% significance level.						
